# A mindfulness-based intervention for Substance Use Disorder in a Brazilian vulnerable population: a feasibility mixed method study

**DOI:** 10.3389/fpubh.2024.1381489

**Published:** 2024-10-30

**Authors:** Mayra Pires Alves Machado, Emérita Sátiro Opaleye, Andre Bedendo, Sarah Bowen, Ana Regina Noto

**Affiliations:** ^1^Núcleo de Pesquisa em Saúde e Uso de Substâncias, Departamento de Psicobiologia, Universidade Federal de São Paulo, São Paulo, Brazil; ^2^Department of Health Sciences, University of York, York, United Kingdom; ^3^School of Graduate Psychology, Pacific University, Forest Grove, OR, United States

**Keywords:** mindfulness, meditation, substance use disorder, vulnerable population, feasibility, outpatient treatment, public health services, implementation

## Abstract

**Introduction:**

Substance Use Disorder (SUD) is a chronic condition that impacts various facets of an individual’s life, and society as a whole. The Mindfulness-Based Relapse Prevention (MBRP) protocol is an innovative intervention that can help to prevent relapse, particularly when used as a post-treatment approach. However, although there is significant evidence of its effectiveness in studies from high-income countries (HICs), there is a dearth of studies examining its feasibility and efficacy in low- and middle-income countries (LMICs). Thus, this study investigates the feasibility of MBRP as an adjunct to outpatient treatment for SUD in a socially vulnerable Brazilian population.

**Methods:**

The study employed a mixed-methods design in eight Psychosocial Care Centers for Alcohol and Drugs (CAPS-ad) in the city of São Paulo, and involved 140 participants, 24 healthcare professionals and 7 CAPS-ad managers. In total, 17 MBRP intervention groups were conducted. The study assessed qualitative indicators of acceptability, demand, implementation, adaptation, integration, and limited efficacy testing through group interviews, in-depth interviews and field diary records. It also included limited efficacy testing of the protocol using a quantitative pre-post pilot study to investigate consumption behavior, using the Timeline Followback (TLFB) assessment method; depression, using the Center for Epidemiologic Studies Depression (CES-D) scale; anxiety, using the state trait anxiety index (STAXI-2); craving, using the Penn Alcohol Craving Scale (PACS); readiness to change, using the Readiness-to-Change Ruler (RCR); and severity of dependence, using the Severity of Dependence Scale (SDS). The qualitative data were triangulated with the quantitative data to comprehensively evaluate the feasibility of the intervention.

**Results:**

The sample comprised socially vulnerable participants with a high dropout rate, primarily due to social factors. Despite facing challenges in respect of regular engagement and initial cultural misperceptions of meditation, the intervention showed positive acceptance and mental health benefits, including impacts on consumption behavior.

**Discussion:**

The study emphasizes the importance of adapting the format of the protocol to better suit vulnerable populations, and to ensure its effective integration into the public healthcare system. Future research should explore protocol modifications, assess its effectiveness in different contexts, and conduct cost-benefit analyses for broader implementation.

## 1 Introduction

Substance Use Disorders (SUDs) are defined by dysfunctional patterns of substance consumption behavior, resulting in impairments across various life domains (American Psychiatric Association, 2013). SUDs significantly contribute to the global burden of disease and are recognized as a worldwide public health issue. Alcohol consumption ranks among the top 10 risks in respect of global mortality, and was the leading risk factor for the global burden of disease in 2019 (1).

SUDs are characterized as chronic conditions, with complex therapeutic management and high relapse rates ranging from 40 to 60% within a year of treatment (2). Cravings, anxiety, depression, anger, and other negative effects are among the primary predictors of relapse (3).

Contemporary psychology has used mindfulness meditation to help to develop awareness and skillful responses to mental reactions that foster stress, dysfunctional behavior, and psychopathology (4). Mindfulness-Based Interventions (MBIs) have been employed in various contexts and populations (5–7). Systematic review studies and meta-analyses on the effects of MBIs in individuals undergoing treatment for SUDs indicate improvements in substance use and abstinence outcomes, reduced cravings, impulsivity, stress and post-traumatic stress, avoidant coping strategies, anxiety, depression, and negative effects (8, 9).

Mindfulness-Based Relapse Prevention (MBRP), and MBI designed to prevent the occurrence and severity of SUD relapse, aims to enhance awareness of high-risk situations that trigger relapse. It also promotes the development of skills to manage negative effects and other discomforts caused by high-risk situations, expands coping strategies, and fosters greater self-care, self-compassion, and a balanced lifestyle (10). Clinical trials have shown the benefits of this protocol in respect of reducing the number of days of use, the amounts of alcohol or other drugs consumed, the risk of relapse compared with standard Relapse Prevention or usual treatment (psychoeducational or twelve-step) (11–13). Studies also indicated improvements in the mindfulness components of acceptance, acting with awareness and non-judgment, as well as reduced cravings and fewer medical and legal problems (11–13).

Although research has advanced in respect of the effectiveness of evaluations in high-income countries, there is a scarcity of studies investigating the implementation and dissemination of MBIs. Moreover, those that have been undertaken were predominantly conducted with WEIRD (western, educated, industrialized, rich, and democratic) populations, which do not represent the majority of the global population (14, 15). Therefore, there is a significant evidence gap in low-and middle-income countries (LMICs) regarding the feasibility of implementing MBRP in more diverse or “real-world” treatment contexts (16).

Brazilian Psychosocial Care Centers for Alcohol and Drugs (CAPS-ad) serve as essential public services, offering outpatient psychosocial care for patients with SUDs. Services include individual and group multidisciplinary psychosocial support, family guidance, crisis management, therapeutic activity groups, harm reduction initiatives, and the CAPS-ad do not require clients to become abstinent to receive treatment (17, 18). The majority of individuals undergoing treatment in these services are adult men of mixed and black ethnicity, who are typically unemployed, single, with only primary education and a low family income. They tend to be polysubstance users, with a predominance of alcohol consumption, and a high prevalence of experiencing and committing violence, thus representing a population with great social vulnerability and disadvantaged socioeconomic conditions (19–23). Combined with cultural disparities, these factors can influence the implementation and efficacy of MBRP in Brazil.

Therefore, the present study investigated, through mixed methods, the feasibility of implementing MBRP as an adjunct, therapeutic, public outpatient treatment for socially vulnerable individuals with SUDs in Brazil.

## 2 Materials and methods

### 2.1 Study design

A mixed-methods study was conducted to investigate the feasibility of the intervention in the CAPS-ad following the indicators of acceptability, demand, implementation, adaptation, integration, and limited efficacy testing as described by Bowen et al. (24), with simultaneous collection of quantitative and qualitative data that was integrated into the analyses.

The qualitative approach investigated the indicators of acceptability, demand, implementation, adaptation, and integration. Data were collected in field diaries throughout the implementation of the MBRP program in the studied services, and, at the end of each intervention group, semi-structured interviews were conducted with managers, professionals, and service users. Limited efficacy testing of the MBRP through a pre-post intervention pilot study was undertaken to evaluate the benefits of the program for service users in respect of substance use and mental health outcomes.

### 2.2 Setting

The study was conducted in eight CAPS-ad in the city of São Paulo (30.7% of the city’s services) from 2016 to 2018. Services were selected for convenience considering geographic variability and characteristics that favored sample heterogeneity. The number of participating services in the research was determined by theoretical saturation from the qualitative analyses (25) in which the data collection was concluded when the information started to become redundant or repeated, even with the inclusion of new participants and services, as detected through a floating reading of the transcripts of the conducted interviews and fields diaries.

### 2.3 Sample

The sample comprised three categories: managers, professionals, and service users. The managers (*n* = 7) had the sole inclusion criterion of being interested in participating in the research and contributing to the investigation of different indicators in respect of the feasibility of the intervention. Their backgrounds were in psychology, psychiatry, and occupational therapy. They participated in the initial stage in which the project was presented to the CAPS-ad according to procedures described in detail below, and were interviewed after the conclusion of at least one intervention to better understand how it fitted into the service operation, to give their perceptions of the impact of MBRP on patients and the CAPS-ad itself, as well as their views on the possibility of implementing MBRP.

The professionals (*n* = 24) were either referred by the managers or volunteered after participating in a project presentation by the research team, with the inclusion criterion of being responsible for the therapeutic process of the service users. They were from the fields of psychology, psychiatry, occupational therapy, nutrition, nursing, and social work. During the period of the research, they assisted in integrating the MBRP group into the service’s activity schedule, defining the physical spaces of the groups, referring service users to participate, and were also interviewed at the end of the intervention. Some professionals (*n* = 16) chose to participate in the MBRP groups along with the service users for a better understanding of the intervention. Those who did not participate had expressed an interest in doing so, but could not due to scheduling difficulties because of their workload within the service.

People with SUDs undergoing treatment in the services, here referred to as service users (*n* = 140), were included based on the following criteria: (a) being literate adults over 18 years old; (b) being in treatment at one of the services for at least 1 month; (c) be diagnosed with SUD by the service’s responsible physician; (d) have an interest in the objectives of this study and consented to participate in the 8-week course. Service users presenting conditions diagnosed by the service’s responsible physician that contraindicated mindfulness practices, such as psychotic disorders, severe cognitive impairment, and suicidal ideation, were excluded from the study.

The service users were majority male (85%), aged between 41 and 60 years (63.6%), single (47%), and with education up to high school level (65.0%). Half of the participants reported an income equivalent to a maximum of two minimum wages (approximately 400 US$ monthly when converting from the Brazilian real), with up to three individuals living on this income in 77.4% of the cases. Employment with formal job contracts was reported by only 13 participants (9.3%), with 76 (54.3%) reporting unemployment and 28 (20.0%) being self-employed with occasional informal jobs. Regarding substance use, 59 (42.1%) participants were diagnosed with SUD due to alcohol use and 66 (47.2%) were polysubstance users (among these, the majority consumed alcohol concomitantly with cocaine or crack, or marijuana). The average age at the onset of treatment was 36.2 (±11.9), with 67 (54.9%) undergoing their first treatment at the referred CAPS-ad. Almost one-third of the sample (29.1%) lived in therapeutic housing or shelters during the intervention due to being homeless before the treatment, and slightly more than half of the participants (*n* = 76, 54.3%) were abstinent at the start of the study. Subtracting the current age from the age at first treatment, a variable named chronicity was generated, with an average of 10.0 (±9.6) years. Table 1 presents the characteristics of the service users’ sample.

**Table 1:** Characteristics of the service users’ sample.

	*N* (%)
Age	
19–40	42 (30.0)
41–60	89 (63.6)
60 +	9 (6.4)
Missing	0 (0.0)
Gender	
Male	119 (85.0)
Female	21 (15.0)
Missing	0 (0,0)
Education	
Elementary school	34 (24.3)
High school	57 (40.7)
Above high school	47 (33.6)
Missing	2 (1.4)
Income^*^	
Up to 2	70 (50.0)
3–5	41 (29.3)
Above 5	20 (14.3)
Missing	9 (6.4)
Employment	
Unemployment	76 (54.3)
Informal job	28 (20.0)
Formal job	13 (9.3)
Medical leave/retired	16 (11.4)
Missing	7 (5.0)
Homeless situation	
Yes	39 (27.9)
Missing	1 (0.7)
SUD	
Alcohol	59 (42.1)
Cocaine/crack	11 (7.9)
Cannabis	3 (2.1)
Polysubstance users	66 (47.2)
Medications	1 (0.7)
Abstinence at T0	
Yes	76 (54.3)
Missing	0 (0.0)
Chronicity (years of treatment)	
0	16 (11.6)
1–10	68 (49.3)
11–25	41 (29.7)
26+	13 (9.4)

* Brazilian minimum wages, approximately 200 US$ monthly when converting from the Brazilian real.

### 2.4 Procedures

**Figure 1:**
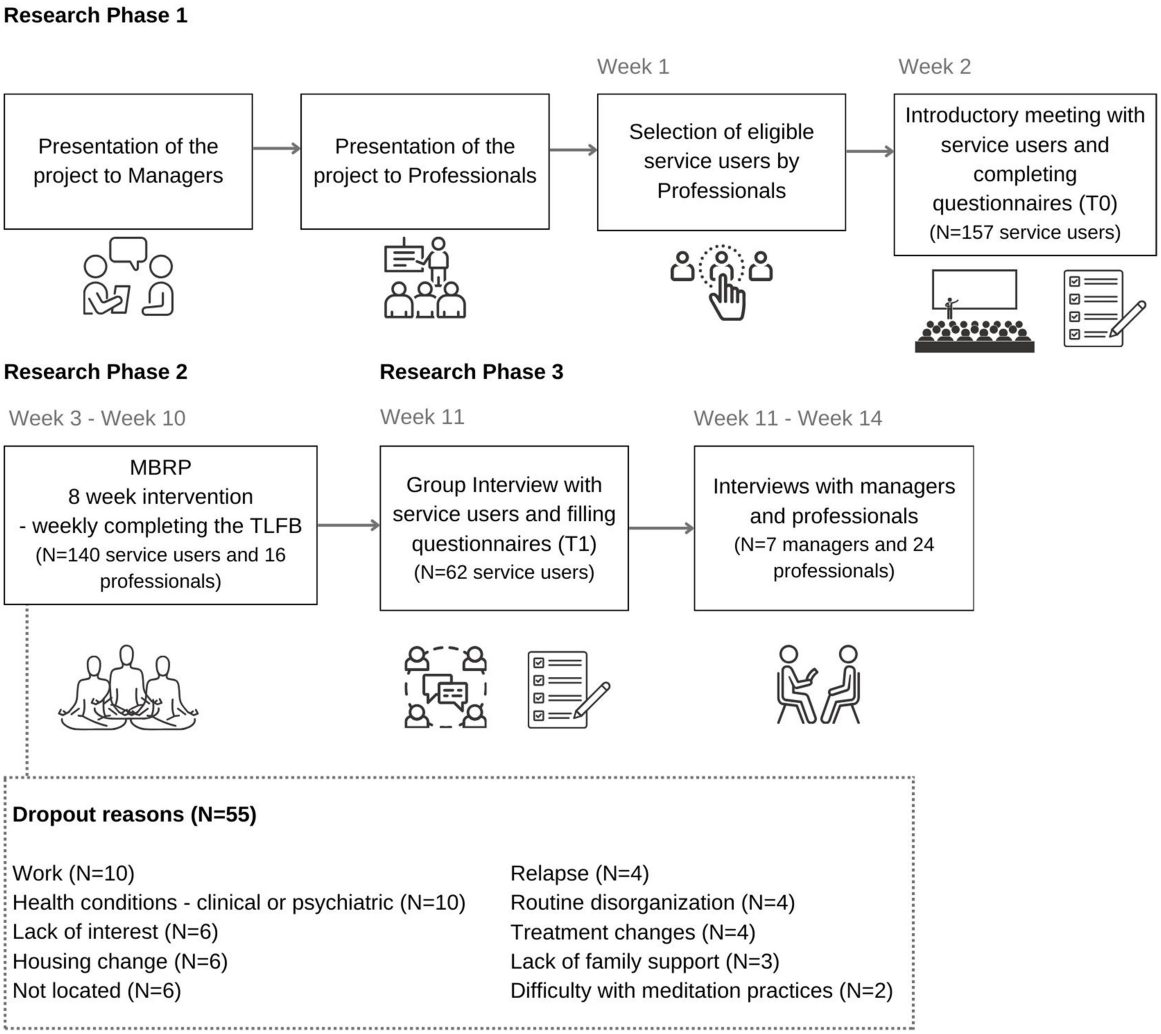
Flowchart of the research procedures.

Figure 1 presents a flowchart of the procedures regarding the simultaneous collection of qualitative and quantitative data over time. In the first phase, visits were made to the services to inform managers and professionals to the intervention and the objectives of the research. Service users were recruited based on medical evaluation and professionals’ recommendations, identified as eligible patients according to the inclusion criteria and invited them to participate in an introductory meeting aimed at presenting the objectives and procedures of the research. From this phase, those who agreed to take part signed an Informed Consent Form, and data collection began with pre-intervention questionnaires (T0). In total, 157 service users completed the pre-intervention questionnaires (time T0) but 17 did not start the intervention and were therefore excluded from the study. With a minimum of four participants, the MBRP intervention groups were offered in each service, with quantitative data collection (substance use) and qualitative data (field diary entries) occurring concurrently. Those who did not attend three consecutive sessions were considered dropouts, which occurred with 55 service users (39.3%). In the week following the intervention, users completed the post-intervention questionnaires (T1) and participated in a group interview. However, 23 (16.4%) who completed the sessions did not attend T1, and no information was gathered on non-attendance. Interviews with managers and professionals were also conducted after the intervention, scheduled according to their availability within the month following the intervention. Data collection is described in more detail below in the sections on the qualitative and quantitative parts of the study.

### 2.5 Intervention

Mindfulness-Based Relapse Prevention (MBRP) is an approach that integrates the Relapse Prevention Model from Cognitive Behavioral Therapy (CBT) with elements from Mindfulness-Based Stress Reduction (MBSR) and Mindfulness-Based Cognitive Therapy (MBCT), and is designed to aid in the relapse prevention of individuals with SUDs (10). It was originally developed to be applied in groups of up to 15 people who are in the maintenance phase—i.e., post-treatment for SUDs—in eight weekly sessions lasting 2 h each, facilitated by a professional specifically trained for this purpose (10).

Between sessions, audio recordings of guided meditation practices are provided, along with informational worksheets and instructions for activities to be conducted at home (10). Participants are encouraged to engage in these practices daily throughout the week, fostering the development of autonomy and enhancement of the skills addressed in the sessions (10).

A total of 17 groups were conducted by the same professional (an occupational therapist) to ensure a more consolidated perception of the feasibility assessment process, and when possible, co-facilitated by a psychologist. Both facilitators had substantial meditative practice and specific professional training in MBRP. Professionals and managers were invited to participate in the intervention alongside service users.

### 2.6 Qualitative data

#### 2.6.1 Data collection

Three qualitative data collection techniques were employed: group interviews, in-depth interviews, and field diary notes. The group interviews were conducted post-intervention with service users without the presence of professionals, managers, or intervention facilitators by a facilitator from a trio of higher education professionals trained both in MBRP and in conducting group interviews, using a semi-structured interview guide to ensure coverage of the indicators investigated according to Bowen et al. (24). The facilitators of the group interviews had no prior contact with the service users. The group interviews lasted, on average, 60 min.

In-depth interviews were conducted with managers and professionals using semi-structured scripts by the principal researcher of this study. These interviews occurred in the month following the completed intervention, averaged 43 min in duration, and were conducted in person at the services on days and times chosen by the professionals, in a private setting. Both group and in-depth interviews were audio-recorded for later transcription and analysis. The semi-structured scripts of group and in-depth interview may be accessed at Supplementary material.

Weekly field diary notes were also made during the intervention to record specific details that might influence the feasibility of implementing MBRP in the services. After each service visit and session facilitation, the researcher recorded notes, perceptions, comments from participants, and any occurrences that could be relevant for a better understanding of the research objective.

#### 2.6.2 Indicators

Table 2 presents the investigated indicators with their respective definitions, measures, source, and data collection procedures.

**Table 2:** Indicators, measures, data source and procedures.

Indicators and definitions	Measures	Data source	Procedures
**Acceptability** *Exploring acceptability of MBRP among CAPS-ad users and professionals.*	User satisfaction with MBRP	Service users	Group interview
	Intention to continue practices post-intervention	Service users	Group interviews and an analog scale at T1 regarding continuation intentions
	Facilitators’ perceptions of acceptability among service professionals and users	Researcher	Field diary notes
	Appropriateness of MBRP as adjunct to outpatient treatment	Managers, professionals, and service users	Interviews (managers and professionals) and group interview (service users)
**Demand** *Investigating demand for MBRP implementation in services.*	Perceptions of MBRP’s positive/negative effects	Managers and professionals	Interviews and field diary entries
	Previous interest and intervention adherence	Service users	Analog scale on interest (T0) and dropout rates
	Perceived demand by professionals and users	Managers, professionals, and service users	Interviews (managers and professionals) and group interview (service users)
	User likelihood to use intervention for behavior change	Service users	Group interview
**Implementation** *The extent and aspects of MBRP implementation in services as it is originally proposed.*	Degree of execution, including success or failure	Researcher	Estimation of the percentage of completion of all stages of the intervention, based on field diaries
	CAS-ad positive/negative factors affecting implementation	Researcher, managers and professionals	Field diary notes and interviews
	Barriers to adhering to MBRP activities	Managers, professionals, and service users	Interviews (managers and professionals) and group interview (service users)
	Perception of the positive or negative effects of the intervention on users	Managers, professionals, and service users	Interviews (managers and professionals) and group interview (service users)
**Adaptation** *Assessing the need for MBRP adaptations in the Brazilian outpatient treatment context for SUDs.*	Need for changes in MBRP format, session structure, and activities	Researchers, professionals, and service users	Field diary notes, interviews (professionals) and group interview (service users)
**Integration** *Exploring the possibility of integrating MBRP into existing service activities*	Suitability for existing infrastructure	Researcher, managers and professionals	Field diary notes and interviews
	Suitability for service objectives and culture	Researcher, managers and professionals	Field diary notes and interviews
	Compatibility of MBRP with CAPS ad activity schedules	Managers and professionals	Interviews
	Manager and professional interest in integrating the intervention	Managers and professionals	Interviews
**Limited efficacy testing** *Limited test of the intervention efficacy*	Pre-post-impact of the intervention on consumption behavior and mental health.	Service users	Self-report questionnaires and scales

#### 2.6.3 Qualitative analyses

The qualitative data from in-depth interviews and group interviews underwent literal transcription from audio recordings. These transcriptions and field diary notes were inputted into NVivo® software. Interviews were coded using alphanumeric codes as described in Table 3.

**Table 3:** Description of alphanumeric codes.

Reference	Code	Example
Group Interview with service users	IG + group number	IG_8: group interview in group 8
Interview with professionals	IP + service number + profession^*^ + gender^¥^	IP_6_EN_M_TO_F: an interview with a male nurse and a female occupational therapist from service 6; IP_4_TO_F: interview with a female occupational therapist from service 4
Interview with managers	IM + service number + gender^¥^	IM_5_F for an interview with a female manager from service 5

* Profession: SW, Social worker; NU, Nurse; N, Nutritionist; PS, Psychologist; PC, Psychiatrist; OT, Occupational therapist.

¥ Gender: F, female; M, male.

Based on the feasibility indicators defined by Bowen (24) as themes, a floating reading was conducted to define categories linked to these themes, followed by coding and content analysis (26). Two researchers independently coded all transcripts, with sporadic meetings for triangulation to minimize subjective perceptions of a single researcher. Divergences were discussed with a third researcher.

### 2.7 Quantitative data

#### 2.7.1 Data collection

Service users completed quantitative measurement instruments using pen and paper at baseline (T0), 1 week prior to starting the MBRP group. Data collection occurred at the services, collectively, in the presence of one or two researchers and without third parties. From the beginning of the MBRP group, weekly monitoring of substance use behavior was conducted using the Timeline Followback Method Assessment (TLFB) questionnaire. Following the intervention’s conclusion, the scales for quantitative measures (T1) were repeated in the same format as T0, except for the pre-MBRP analog-visual scale of interest, which was replaced by another scale to investigate the likelihood of continuing practices post-intervention.

##### 2.7.1.1 Measures

Sociodemographic data, substance use information, and prior interest in MBRP participation (an analogic-visual ruler that varied between 0 “no interest” and 10 “very interest”) were collected via a researcher-developed questionnaire and medical record reviews. For other quantitative outcomes, the following scales were applied:

*Substance Use Behavior*: The Timeline Followback Method Assessment (TLFB) (27) was used, which records variables in respect of abstinence duration; the period, frequency, and quantity of substance use in relapse and/or lapse episodes over the last 15 days (T0 or T1), and was completed weekly during the intervention with information on substance consumption during the last 7 days.

*Craving:* Craving was evaluated using the Penn Alcohol Craving Scale (PACS), a self-report scale consisting of five items, scored from 0 to 6, to assess the intensity, frequency, and duration of alcohol craving, as well as cravings experienced in the last week and difficulty in resisting if the substance is available (*α* = 0.92). The scale was adapted for use with all substances by including terms specific to the “substance in question” (28).

*Anger Expression*: measured using the Anger Expression Index (AEI), a comprehensive index derived from four subscales of the State–Trait Anger Expression Inventory (STAXI-2). These subscales assess Anger Control-In (managing contained anger and regulating felt anger, such as trying to calm down when irritated) and Anger Control-Out (controlling anger felt in a way to prevent its expression toward others or objects in the environment), as well as Anger Expression-In (anger directed inward, kept or suppressed) and Anger Expression-Out (anger expressed toward other people or objects in the environment). Each subscale comprises eight items on a Likert scale ranging from 1 (“never”) to 4 (“almost always”) (29).

*Depression Symptoms*: The Center for Epidemiologic Studies Depression Scale (CES-D) was used, a self-report instrument comprising 20 items. Participants indicated the frequency of experiencing depressive symptoms in the past week on a 4-point Likert scale ranging from “rarely or none of the time” to “most or all of the time” (30). In individuals with SUDs, scores above 24 points indicate the presence of depression (31).

*Anxiety Symptoms:* The assessment was conducted using the State Trait Anxiety Inventory, specifically the Trait Subscale (STAI-T). This subscale consists of 20 items on which participants rate in respect of how they generally feel on a Likert scale ranging from 1, “almost never,” to 4, “almost always” (32).

*Readiness for Change:* Measured using the Readiness Ruler, an analog visual technique that rates on a scale from 0 to 10 how ready an individual feels to change their behavior related to substance use. On this scale, 0 signifies “not prepared to change,” and 10 indicates “already changing” (33).

*Severity of Dependence:* This was assessed using the Severity of Dependence Scale (SDS), which consists of five items on a 4-point Likert scale ranging from 1, “never/almost never,” to 4, “always/nearly always” (34).

#### 2.7.2 Quantitative analysis

The questionnaires were double-entered into the RedCap® platform by independent researchers. Quantitative data were analyzed using STATA 14® software. Descriptive analysis was performed to characterize the sample, using means and standard deviations for continuous variables and frequency for categorical variables. Exploratory analyses were conducted to evaluate differences between T0 and T1 as potential indicators of MBRP’s limited efficacy. To identify possible changes post-intervention, subjects were first classified as having either fixed or random effects based on residual analysis from scatter plots and the intraclass correlation coefficient (ICC). ICC only supported the use of random effect for the model predicting the percentage days of cocaine or crack consumption (ICC: 0.602). Subsequently, Mixed Linear Models (MLM) were employed for subjects with random effects, and Generalized Estimating Equations (GEE) (35) for those with fixed effects. Both methods are part of the Generalized Linear Models preferred in longitudinal analyses for considering individual variance over time, and include all subjects who started the intervention, adhering to the intention-to-treat analysis principles. Age and chronicity of SUD were controlled for in all analysis. The data were triangulated with qualitative reports of benefits.

Specifically for substance use behavior, TLFB data were grouped into four time points: 15 days prior to data collection (T0), from T0 to week 4 of the intervention, from week 5 to week 8 of the intervention, from week 8 to T1. For each of these periods, the percentage of days of heavy alcohol consumption, percentage of days of cocaine/crack consumption, average consumption of standard doses of alcohol, and average consumption of marijuana cigarettes were calculated.

### 2.8 Ethical aspects

This research adhered to the Declaration of Helsinki and was approved by the UNIFESP Research Ethics Committee #1.346.744 and the Research Ethics Committee of the Municipal Health Department of São Paulo #1.370.371. Participants were informed about the voluntary nature of participation, anonymity, and the freedom to withdraw at any time without detriment. Participation was confirmed by signing an Informed Consent Form. No financial incentives were offered to participants, and they were informed about potential risks. To promote intervention sustainability, MBRP training was offered free of charge to a professional from each service at the end of data collection.

## 3 Results

Qualitative indicators were categorized into three main themes based on content analysis: (1) Acceptance and Demand, (2) Implementation and Adaptation, and (3) Integration. Findings from the quantitative data of limited efficacy testing were triangulated with the qualitative measure of “perception of positive or negative effects of the intervention on users” within the Implementation indicator, and are presented together as a fourth theme named “Benefits of MBRP as a complement to usual SUD treatment.”

### 3.1 Acceptance and demand

Three categories were identified: (a) pre-group receptivity and barriers; (b) satisfaction and adherence; (c) incorporation into life.

#### 3.1.1 Pre-group receptivity and barriers

Initial receptivity to the MBRP program was higher among service managers and professionals than among service users, with the latter group expressing more doubts. The service users’ receptivity to the proposal varied from curiosity to refusal. Among those attending the introductory meeting, there was significant prior interest in participating in MBRP, with an average score of 8.83 on a visual-analog scale, with over half (54%) scoring the maximum.

*At first, I found it very strange, I think the lack of [experience with] practice itself. The first time is always different and we have to wait to see if it makes sense. Does it really make sense? (IG_11)*

Several pre-group barriers were identified, primarily related to cultural factors and social vulnerabilities. Professional sensitization was key in overcoming these barriers to facilitate user engagement. Meditation was often associated with “zen” aspects, perceived as belonging to a more privileged class, linked to religions, and considered difficult and achievable only by monks.

*So there are also some things from this imaginary, this thing of Yoga, Zen, eating fruit, that’s not my thing. (IP_2_OT_F)*

Another aspect mentioned was the perception that the practice of mindfulness meditation is aimed at people who are already being calmer and have a healthier lifestyle, therefore, incompatible with the profile of people who use substances such as alcohol and cocaine, in addition to the users’ natural resistance to extra activities in the service.

*This is not for me, it’s too zen, it’s too still, I’m not like that, I’m too fast, I’m too electric, I won’t be able to sit still. (IP_2_OT_F)*

Another barrier primarily reported by professionals was the increased vulnerability of some service users, particularly during acute phases of Substance Use Disorder (SUD), in which they are unable to sustain abstinence for extended periods. This condition severely impacts their comprehension, reasoning, ability to structure routines, and adherence to any other service activities, alongside significant social and clinical consequences. Given the lack of aspects of basic needs for subsistence such as food and hygiene, their provision becomes a priority to the detriment of other therapeutic processes.

*I think that these patients [with high vulnerability] are worried about getting these [basic] things and they can't deal with being able to look at them a little bit now. (IP_4_OT_F_PQ_F)*

#### 3.1.2 Satisfaction and adherence

User satisfaction was reported at the conclusion of the MBRP, and was associated with the perceived benefits and contribution to treatment of the program, which will be more thoroughly detailed in item 3.4.

*On the day of the meeting, Friday meditation, I was hoping to leave here feeling lighter to spend my weekend. It was fantastic for me … it even generated an expectation “oh, there’s meditation today”. (IG_16)*

The reports highlight the development of mindfulness components (attention to the present moment and attitudes) and related skills (self-compassion), which in turn facilitated changes in behavioral and emotional outcomes, thereby impacting substance use outcomes and relapse prevention. In this context, relapse prevention itself appeared to be just one element of change among many others significant to each individual’s life.

*I learned to have a relationship with my body. Because the fact that I breathe and feel my legs, my upper and lower limbs and have that relationship, that's what… makes me get in touch, that I have… how do you say… more self-control. (IG_4)*

Regarding adherence, the majority of users did not drop out of the intervention, with 85 (60.7%) individuals completing it. It was noted that aspects of daily life and lack of social support (such as housing changes, work, lack of support, family matters, routine disorganization, and the need to study for exams) accounted for 41.7% of the reasons for dropout. Relapse and worsening of other psychiatric conditions represented 16.4%, while dropouts due to lack of interest or difficulty with the intervention totaled 14.5%.

#### 3.1.3 Incorporation into life

Service users appeared able to incorporate mindfulness skills into their daily lives. However, part of the protocol involves performing formal meditation practices between sessions, which was a significant difficulty for some health users.

Several challenges were encountered: lack of audio equipment for guided practices; inadequate physical space or unfavorable housing conditions, homelessness, embarrassment and lack of family support, forgetting to practice until it becomes a habit, and inherent challenges of meditation practice such as restlessness and drowsiness.

*For me the lack of support. It's just that my sister (…) If she sees me practicing, she gets angry. So I have to do it when she is not there or when she has already gone to sleep. (IG_5)*

The average score for the likelihood of continuing practices post-intervention at T1 was 7.95, supporting the reports of a strong intention to continue with the practices.

### 3.2 Implementation and adaptation

On average, 98.9% of the protocol activities were applied in the groups, achieving a good coverage rate. However, field diary records suggested some modifications to the original protocol for better user absorption, including changes in the order of some activities.

Barriers to treatment at CAPS-ad were identified, impacting the implementation of the protocol. These included the profile of the socially vulnerable population, low treatment adherence, or precarious housing conditions. Field diary notes included reasons for absences such as lack of money for transport, the need to work to buy food, and less frequently, absences due to substance use episodes. Specifically, among women, there were reports of greater stigma and difficulty in joining a treatment predominantly attended by men, along with higher social demands in home management and childcare.

*On the one hand, the issue of gender is being discussed. There are women who drink at home, who are ashamed and (…) have more difficulty connecting to the service. (IP_2_PS1_F)*

Challenges were also encountered with the infrastructure of CAPS-ad. Often, there were significant external noises disrupting user concentration, or the rooms were too small for activities like walking or movements, with a lack of chairs and whiteboards for psychoeducational activities. Upon concluding the intervention, participants showed a keen interest in continuing, yet also expressed insecurity about their ability to practice autonomously. This led to a suggestion for an adaptation involving two weekly meetings with shorter durations and more sessions.

*Do you know what my fear is? It's like, after it's over, a month goes by and we lose (…) It would be nice to reduce the hours and put it twice a week. (IG_11)*

According to field diary records, during the intervention sessions, service users mentioned that participation required more concentration, discipline, and life organization than they initially thought possible. They also commented on the complex language and faster pace of content delivery, which hindered appropriate assimilation, and the session duration being longer than they were accustomed to. The use of whiteboards and handouts posed challenges for illiterate individuals or those with cognitive impairments.

Field diary records indicated that participants who were homeless, living in shelters, or in assisted housing lacked resources to listen to practice audios but revisited printed handouts given in each session to learn the covered themes. Thus, the creation and provision of simplified printed materials with basic instructions for each practice were suggested. Additionally, finding suitable or safe spaces for meditation practices was a difficulty.

*Where I live there's no way to do it. I try to do… I live with ten people, so you don't have much freedom, you don't have privacy. (…) So I tried to do it my way, but not at home like that. (IG_11)*

Associated with this, there is also prejudice on the part of third parties that also becomes a challenge.

*Society itself, that is, the environment I frequented in bars… Commenting that a process like this is being carried out here, there are those jokes like “this is a fag thing”, For the real world we live in out there, this is something that is more for the upper middle class. The world itself, class D and E, already sees this as more of a thing… fresh [overly sensitive or delicate]. Then you come and do it and see that it’s nothing like that. That it is cool! (IG_4)*

Field diary records highlighted the impact of events at the services, such as peers’ relapses, deaths, violence, and user upheaval due to changes in treatment policies or staff dismissals. This required the facilitator’s sensitivity to accommodate these issues when raised by users in the group.

The professionals and users identified several key factors for remaining in the intervention despite these barriers, namely: greater availability for treatment; openness, interest, and curiosity about MBRP; being motivated to change behaviors; encouragement from professionals and family; determination to complete started tasks; recognition that MBRP could help, and understanding that change requires individual effort and does not happen overnight. As sessions progressed, increased engagement and a shift in perceptions toward meditation and its benefits were observed.

*How am I going to be able to do this? [thinking to oneself] But I did not give up… At first, I had a lot of trouble getting used to it. The first few times I did it I couldn't wait for it to end. (IG_1)*

*Then when I saw… that I attended the first, the second, then I started liking it… (IG_10)*

### 3.3 Integration

According to managers and professionals, the MBRP is appropriate for the objectives and culture of the services, but some alterations were suggested for better integration, considering socioeconomic conditions, treatment stage, and motivation level, among other factors. There was a consensus among professionals and managers that the MBRP format could be modified to sessions lasting a maximum of one and a half hours, with a greater number of sessions, possibly in an open and continuous format, allowing participants who join the group after its start to catch up. Therefore, it was suggested to reorganize the content into more sessions to reduce the duration of each. Likewise, once all topics were covered, they would restart for new participants and remain open for those wishing to repeat. They believed this would facilitate the appropriation of practices and gradual assimilation of content.

*I think it's all too fast for the content density. If I had the opportunity, I think it would be really cool to add a couple of sessions, I don't know, to be able to work a little more calmly, carefully on the same topics…repeat (…) And regarding implementation, I think it makes a lot of sense to have a group like this at CAPS, I think it makes sense to be a closed group at the beginning, middle and end. (IP_4_PS_F)*

*… they need more time, … They need something more, to spend six months, practicing, (…) I think that if they continued, they would make the most of it. (IP_5_PS_F)*

Regarding infrastructure suitability, although CAPS-ad facilities have spaces for groups, there was often significant noise, which hindered participant concentration. There were also reports of discomfort with the chairs, and a lack of resources such as whiteboards, the ability to print handouts with home activities, mats, and cushions.

*USER 1: Regarding the structure, I think it could be improved a little because there’s a lot of noise. The space could be a much more thoughtful place, with better acoustics, because it's very distracting, right?*

*USER 2: And what I didn't like was the room. Very uncomfortable, a lot of people, I said, I'm going to hit someone here. For me, a very small space like that, a lot of people, we start to get irritated, we even feel sick, dizzy. (IG_11)*

Although there was great interest among managers in integrating the intervention into the service, the lack of human resources was cited as a barrier as the intervention requires a trained professional to conduct the sessions, ideally one with an interest in this type of meditative approach.

*Look, I'll be honest, right now, I can't even think about implementing anything, I need to have professionals in here.” (IM_7_F)*

*But training implies commitment, and I don't know how much people's curiosity about mindfulness would transform into personal commitment. (IM_2_M)*

*So, I think having therapists capable of working with the group in terms of more public policy would be the first big challenge. Yeah… I think there would also be another big challenge that we already mentioned, which is people's lack of knowledge. (IM_2_M)*

### 3.4 Benefits of MBRP as a complement to usual SUD treatment

Health users reported increased focus and recognition of their present moment, heightened awareness of their needs and goals, awareness of physical and emotional discomforts due to various triggers, and a stronger connection with their body. Among the attitudes of mindfulness, there were reports of increased curiosity and openness, reduced judgment, and greater kindness, acceptance, and detachment (such as not engaging in interpersonal conflict or clinging to emotions or thoughts). There were also accounts of developing greater self-compassion, a sense of connection with one’s humanity, and a recognition of shared humanity, acknowledging that humans share common needs and are susceptible to suffering. The reports indicated that MBRP not only helped reduce relapse risks but also enhanced protective factors (further information may be accessed at Supplementary material).

*It's helped me a lot, it's clarified a lot of things in my life, it's… including stopping the need to fill a void that has always existed, you know? Meditation helped me to embrace myself, to get closer to myself, you know, to self-love. (IG_16)*

It was also identified that the benefits could also be noticed by their family members. According to professionals and managers, they also noticed an improvement in adherence to treatment, in assuming greater protagonism, even in more refractory users who have been following them in treatment for a long time.

*He has become much more committed to his treatment. I notice that he is calmer, less anxious and speaks more naturally. And he used to say that before he went to sleep at night, his head was racing a mile a minute. He says that since he started doing this practice, he is able to think less, and is able to let go and go to sleep. (IP_8_PS_F)*

Specifically in relation to drug consumption behavior, users reported that they began to have greater recognition of triggers and the discomfort generated and, upon recognition, they seemed to be able to cope with challenging sensations and emotions in a more skillful way instead of acting on impulse in an attempt to avoid or immediately alleviate any discomfort. For this reason, professionals and managers recognized that MBRP was not only a tool to help prevent relapses and lapses, but was also useful in helping with personal reorganization after the lapse/relapse, reducing consumption and even for harm reduction actions.

*I have a little more self-knowledge. I understand myself as I am feeling in a given situation. When there is a trigger that I have, a craving or extreme desire, I can notice that my hand starts to sweat, … they get hot, start to itch, it's a sign that I recognize now… oops, I have to be careful. (IG_11)*

**Figure 2:**
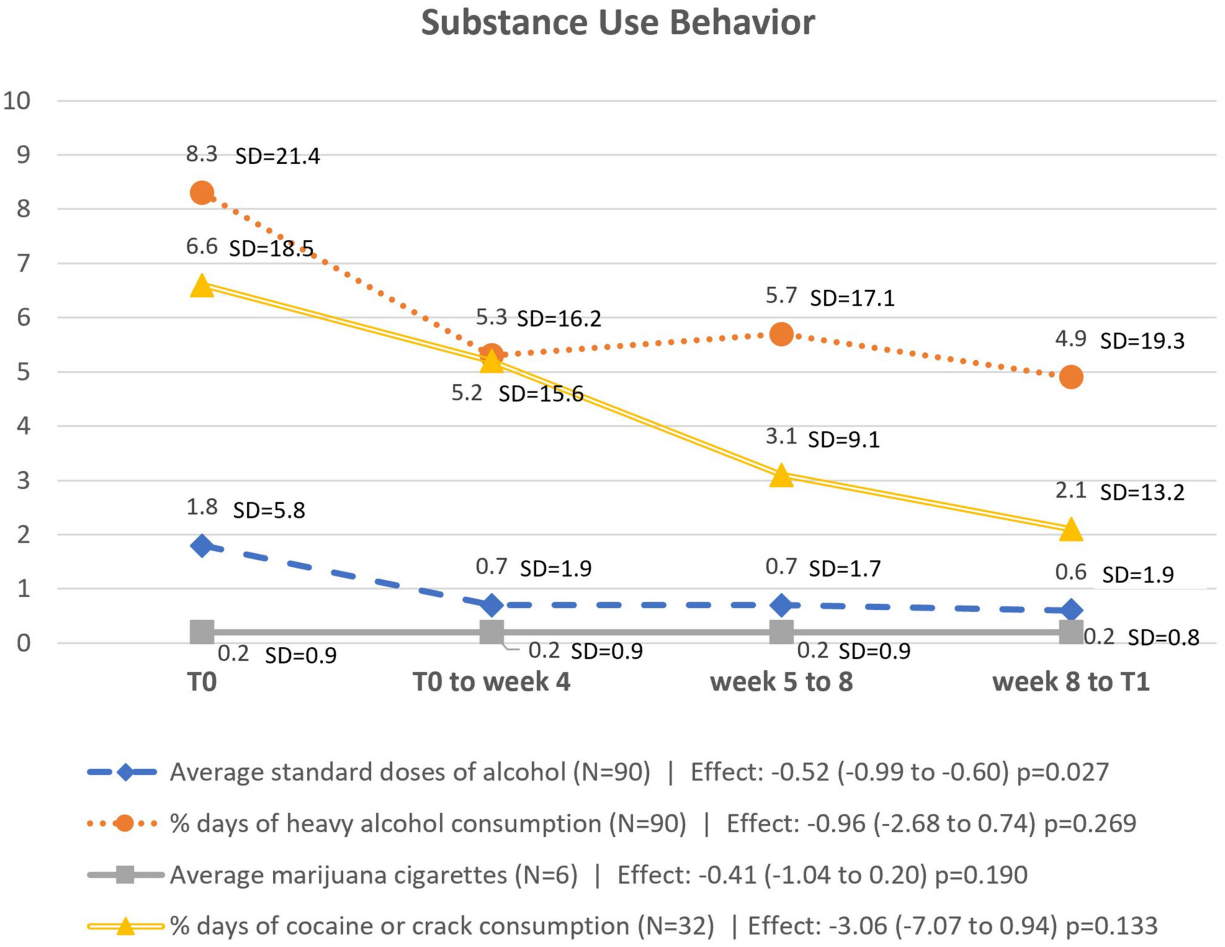
Graphical representation of substance use behavior outcomes over time.

When triangulating the reports with quantitative data, it was observed that there was a statistically significant reduction in the average consumption of standard alcohol doses. Although it is possible to observe a reduction in the percentage of heavy alcohol consumption and cocaine/crack use days over time, this reduction was not statistically significant (Figure 2).

Regarding other mental health outcomes (Table 4), there was a statistically significant reduction in symptoms of anxiety, depression, and craving, as well as in the expression of anger and the severity of dependence. However, it cannot be stated that these reductions were solely due to the MBRP, given the absence of a control group.

**Table 4:** Mental health outcomes over time (*N* = 140).

	T0	T1	Effect^a^
	*M* ± DP (*n*)	*M* ± DP (*n*)	*b* (95% CI)
Dependence severity	15.0 ± 2.9 (135)	13.0 ± 3.8 (62)	−1.9 (−2.7 to −1.1; *p* = 0.000)*
*Missing n (%)*	5 (3.6)	78 (55.7)	
Readiness to change	8.2 ± 2.0 (140)	8.5 ± 1.80 (45)	0.14 (−0.4 to 0.6; *p* = 0.609)
*Missing n (%)*	0 (0.0)	95 (67.9)	
Depression	24.0 ± 11.0 (137)	19.5 ± 9.5 (59)	−4.9 (−7.2 to −2.70; *p* = 0.000)*
*Missing n (%)*	3 (2.1)	81 (57.9)	
Anxiety	49.9 ± 10.4 (128)	44.6 ± 11.6 (59)	−5.3 (−7.4 to −3.3; *p* = 0.000)*
*Missing n (%)*	12 (8,6)	81 (57,9)	
Craving	11.6 ± 7.9 (137)	9.6 ± 8.4 (62)	−2.2 (−3.8 to −0.5; *p* = 0.009)*
*Missing n (%)*	3 (2.1)	78 (55.7)	
Anger expression	43.2 ± 14.9 (131)	40.0 ± 15.5 (62)	−4.0 (−6.4 to −1.7; *p* = 0.001)*
*Missing n (%)*	9 (6.4)	78 (55.7)	

a Adjusted for age and chronicity.

*p < 0.05.

There was a consensus among the service users that there were no harms associated with the intervention, although they mentioned difficulties with certain practices that heightened exposure to challenging emotions. Four participants reported adverse behaviors attributed to the practices, including substance use and feeling unwell during a meditation practice.

*I think I had one negative perception and it was punctual, which was the crisis I had, because of the mind that I couldn't keep up with, I tried and tried, the agony began. I couldn't stay (in the group) it started to give me a phobia and I left… But otherwise, I think I only had to gain. In knowledge… (IG_11).*

Service users, professionals, and managers reported that the MBRP contributed to the service. MBRP seemed to equip both users and the professionals who participated in the intervention with practical tools to use in their personal lives and in respect of treatment. It was noted that the program helped to shift the focus away from “drugs” to broader life aspects that impact substance use behavior. Some professionals highlighted its role in making emotional issues, experiences, and challenges tangible. Users added that MBRP went beyond the existing therapeutic groups and medication treatments, and also provided low-income individuals the opportunity to experience an intervention typically perceived as being for those with higher incomes.

*I've been here for a long time doing analysis and group work, and for me the change in focus was very important, because we changed our focus from talking a lot about addiction problems, and other problems, to thinking a lot about the here and now, about doing practice, which helps us… helps us to change, to be different from what we used to be, and seeing ourselves as different from who think we are. I think this treatment serves as a tool. (IG_8)*

## 4 Discussion

The results of this study suggest that implementing a Mindfulness-Based Relapse Prevention (MBRP) program in CAPS-ad for socially vulnerable populations is feasible, with good acceptance among managers, professionals, and service users. It also indicated that potential treatment demands for SUDs could be met through this intervention. However, MBRP may not be suitable for individuals highly identified with consumption, those with unstructured routines, or with unstable comorbidities such psychotic disorders. While feasible in its proposed form, the MBRP program requires adaptations, including format changes, to better fit CAPS-ad and address the needs of those with the greatest social vulnerability. Given the profile of CAPS-ad patients, the structured format of MBRP (closed groups of eight sequential weeks, 2-h sessions, structured content) may be demanding. The challenges and barriers to integrating the program into the service, and expanding it to other CAPS-ad were identified, the main barrier being professional training.

This is the first study to evaluate the feasibility of implementing MBRP in a real-world scenario in the public health system of a low-and middle-income country using mixed methods. In addition to triangulating quantitative and qualitative data, this study employed triangulation within the qualitative domain, utilizing various sources and data collection methods. Another strength of the study was the diversity and number of services investigated, providing a broader view of intervention dissemination. However, all services were located in São Paulo, thus there is a need for implementation studies in other Brazilian cities with different cultural and socioeconomic backgrounds. Another limitation was the absence of a control group for quantitative analysis, restricting inferences about the intervention’s effect on service users. Additionally, the focus on a highly vulnerable population with limited access outside of CAPS-ad tenure constrained follow-up after the intervention and tracking of users who dropped out. Given the context in which this study was taken, there are numerous variables that could impact treatment and potentially confound inferences about the effect of MBRP, such as participation in other therapeutic activities within the service, support for treatment that promotes adherence, rapport with service professionals, as well as issues related to housing, family, employment, financial resources for transportation, substance use history, potential impairments due to substance use, and the presence of comorbidities. This study contributes to identifying these potential confounders to guide future research, particularly those testing the effectiveness or efficacy of MBRP in these services.

### 4.1 Adherence and barriers

Participants and professionals from the services reported a number of factors that impacted adherence to the MBRP program, including a bias against meditation, leading to “disidentification” with the approach and serving as an initial barrier. Those who started the program generally had a negative first impression but remained due to motivations such as wanting to change, curiosity, discipline, and internal availability. Concentration difficulties, agitation, sleepiness, activity duration, mind wandering, and facing challenging experiences also seemed to affect adherence. However, once these issues were overcome, significant benefits were perceived. However, there remained concerns about maintaining practices and benefits post-intervention.

A systematic review (36) on adherence barriers and facilitators for individuals with chronic conditions to Mindfulness-Based Interventions (MBIs) highlighted practical factors (scheduling conflicts, session duration, family issues, etc.), motivation for change and practice, and participants’ clinical and demographic characteristics. Less prevalent factors were a lack of connection with other participants, credibility, the perception of the intervention, and difficulty with the intervention content. Thus, many barriers to MBI adherence are external to the intervention itself but need to be addressed to ensure effective implementation. Moreover, these barriers may be correlated, where an external challenge, like a lack of family support, intersects with a difficulty in observing thoughts, thus impacting motivation. External factors include issues such as childcare, transportation, and prioritizing subsistence needs, and are frequently most prevalent in vulnerable populations.

Overall, MBRP seems highly beneficial for aiding the rehabilitation of individuals with SUDs in CAPS-ad treatment. However, the current way the MBRP is proposed demands a lot from this population, particularly in terms of discipline, time investment, and internal readiness for awareness and change. Participants suggested format changes to overcome this, which align with the findings in the literature, including a study on a rolling format (16, 37–40), in which MBRP was implemented in an inpatient context with eight sessions divided into two weekly one-hour meetings. The program was well-received by the participants (40), and improvements in mental health outcomes, craving, mindfulness traits, and self-compassion, were recorded, even with participation in only two MBRP sessions in this format (40).

The suggestions for adaptations in the structure of MBIs in the current study are similar to those that have been mentioned in previous studies with clinical populations (36, 41) to better meet the demands of each population. They emphasize the importance of instructors trying to: minimize external factors, combine the programs with motivational aspects or even motivational interviewing, address mindfulness attitudes more explicitly when dealing with meditation challenges, openly discuss external and internal barriers to practice, and promote broader awareness of meditation as a complementary evidence-based practice.

Challenges such as mind wandering, agitation, and sleepiness are inherent to the meditation process and anticipated in MBRP, and have also been identified in other treatments for various mental health conditions (10, 38). However, individuals with SUDs may have a particularly lower tolerance for challenging states, and use substances to relieve or avoid discomfort (42, 43). Developing an internal space for awareness, exploration, and increased acceptance appears to be one of the most significant potential behavioral change mechanisms promoted by MBRP (10, 44). It might be necessary to allow more time for instructor-led practice, and to offer content more gradually to develop this skill sustainably over time with this population, which may mean investigating the possibility of extending the number of meetings—which seems feasible given that participants adhering to treatment stay in CAPS-ad for an average of 6.4 months (45).

A longer period also seems necessary for integrating mindfulness practices into routines and developing new habits. To facilitate this, activities between sessions are encouraged, with the distribution of handouts and audio-guided practices (10). Research indicates a direct relationship between home practice and benefits, so special care should be taken with this population, considering reports from the participants about not having a home, or electronic equipment, which constitute significant barriers to the program (39, 46). Possible alternatives include providing written materials with simplified instructions, simplifying practice instructions for memorization, exploring options for personal practice spaces within the service, utilizing community locations and resources for social reintegration, and encouraging informal practices.

Cultural and language adaptation in materials and sessions is crucial due to the comprehension difficulties reported by participants. Since cultural and language compatibility between therapists and community mental health treatment recipients is linked to better treatment retention and clinical outcomes, MBRP must make necessary adaptations while maintaining protocol fidelity (37, 39, 47). Adaptations should be made both superficially, by using appropriate examples and language, and structurally, which may mean including more self-compassion practices in stigmatized contexts (39), or even to move “Urge Surfing” practice from session 2 to session 3 due to the excessive amount of content relating to triggers covered in session 2, allowing participants to absorb it gradually. Some of the terms commonly used in MBRP that were not so familiar to participants were “triggers,” “relapse,” “risk situations,” “cravings,” “loving-kindness,” “compassion,” and even “kindness.” An alternative would be for the teacher to familiarize themselves with similar terms that are more easily understood by participants, or when it is necessary to use the same term, to provide psychoeducation and ensure alignment of understanding with participants beforehand. The use of overly complex language should be avoided throughout the session, even when naming body parts during the body scan practice. Additionally, between sessions, participants are encouraged to fill out home practice diaries, which is not culturally usual for them.

### 4.2 Future directions

Futures studies are needed to investigate the effectiveness of MBRP within this context, including control groups. Further research is needed to: investigate the expansion of MBRP to other Substance Use Disorder (SUD) treatment contexts such as inpatient community therapy settings; assess the open and continuous format in outpatient contexts; study the cost-effectiveness of implementing MBRP in public health networks; and evaluate MBRP expansion not only for adults with SUDs in CAPS ad but also for other populations (e.g., adolescents, people with other mental health conditions such as anxiety and insomnia, or those seeking mental health promotion, well-being, and balance) and other contexts (e.g., drug use prevention in schools, businesses, etc.). Future pragmatic controlled trials investigating the effectiveness of MBRP in these healthcare settings, which include control groups allowing for comparisons and inferences regarding the effect of MBRP on treatment outcomes, are also needed. Some of the hypotheses that can be tested include the effect of MBRP on outcomes related to substance use, and also on the benefits identified in qualitative reports that are secondary to substance use itself, such as interpersonal relationships, self-care, self-esteem, self-compassion, engagement in protective activities, and social reintegration. More robust longitudinal studies could even investigate if any of these outcomes mediate the possible impact of MBRP on consumption behavior. Additionally, the professional training process for implementing the intervention should be evaluated.

Furthermore, traditional quantitative methods for testing the efficacy or effectiveness of MBRPs may not fully capture the benefits reported by patients and professionals, or perceived clinical improvements. This issue is not exclusive to MBRPs, but also applies to MBIs in general and other Complementary and Integrative Health (CIH) practices. Challenges include conducting methodologically rigorous research with this specific, vulnerable and socioeconomically unstable population (often lacking in basic needs such as housing) and the adherence difficulties encountered in any therapeutic approach, especially in real-world contexts where financial compensation for research participation is not allowed, as in Brazil. In this light, pragmatic studies, quasi-experimental designs, and mixed methods appear to be the most appropriate alternatives (48–51).

To enhance engagement and retention in future interventions, the involvement of service professionals themselves is necessary, as they are already trusted by participants and can encourage them to participate and stay in the intervention. Additionally, future research should consider aspects that, although not objectively defined, have been identified as impacting intervention attendance. These include greater routine disorganization, difficulties in accessing transportation, the need to work informally at times that may conflict with intervention schedules to ensure subsistence (i.e., less social assistance for basic provisions such as food), regularly attending the service while intoxicated by substance use, and low motivation for behavior change.

### 4.3 Conclusion

The results of this study suggest that implementing MBRP in outpatient treatment settings for SUDs in low-and middle-income countries is feasible, but some adjustments are necessary. It was positively received by managers, professionals, and service users, with prior sensitization of professionals helping to overcome initial barriers, such as meditation bias. High satisfaction with MBRP, intention to continue, and perceived benefits by both users and service professionals were reported. MBRP appears to fit well within the organization and culture of the services, aiding in achieving therapeutic goals and meeting demands, making it a valuable complementary tool. However, numerous challenges arose during the process, highlighting the need for the implementation of certain precautions and adaptations to enhance user adherence and further develop the approach.

## Data Availability

The raw data supporting the conclusions of this article will be made available by the authors, without undue reservation.
