# Supplementary material for: A mindfulness-based intervention for Substance Use Disorder in a Brazilian vulnerable population: a feasibility mixed method study
Source: Front Public Health. 2024 Oct 30;12:1381489. doi: 10.3389/fpubh.2024.1381489 (PMC11557387; doi:10.3389/fpubh.2024.1381489)
Supplement: Supplementary file 1 [file Data_Sheet_1.pdf]

Supplementary Material

MBRP for Substance Use Disorder in a Brazilian Vulnerable Population: A Feasibility Mixed Method Study

Mayra Pires Alves Machado[1], **Emérita Sátiro Opaleye**[1*], **Andre Bedendo**[1,2], **Sarah Bowen**[3], **Ana Regina Noto**[1]

[1] Núcleo de Pesquisa em Saúde e Uso de Substâncias, Departamento de Psicobiologia, Universidade Federal de São Paulo, São Paulo, Brazil

[2] Department of Health Sciences, University of York, York, UK.

[3] School of Graduate Psychology, Pacific University, Forest Grove, OR, USA

*** Correspondence:**
Corresponding Author
emerita.satiro@unifesp.br

1 Semi-structured script of the group interview with service users

1. These activities you've been doing may be very new and different from what you're used to. So, how was it being part of this group?

2. Were you satisfied with the course? Did it meet the expectations you had before starting?

3. Did this course bring any benefits to you? If yes, what were they?

4. Did this course bring any disadvantages to you? If yes, what were they?

5. If this program could help you in any way with what you're seeking for your alcohol or drug use treatment, how would it be?

- In your assessment, does it make sense to do MBRP here at CAPS-ad (in an adjunct manner)? Did it help with other activities you participate in here?

6. What practice did you enjoy the most? Why? And the least? Why?

7. Which practice did you use the most? Why?

8. Were there any negative aspects regarding the course?

- Evaluate more structural issues, such as timing, physical space, etc. (e.g., what timing would be better? What did you think of the two-hour duration? And the 8 weeks?)

- What made participation in the course here at CAPS-ad or the completion of practices at home difficult or easy? How was it in terms of physical environment or other aspects? And at your home, how was it? Were they adequate?

- Any suggestions for course improvement?

9. How was it to have a CAPS-ad professional present during the group? How do you evaluate their participation?

- Did it affect your participation in the group in any way?

- Was it possible to notice any difference in how they work or attend to people here at CAPS-ad?

2 Semi-structured script of the in-depth interview with professionals

BLOCK 1. GENERAL CHARACTERISTICS OF THE SERVICE AND USERS

1. Coverage area - what is it and what are the socioeconomic characteristics?

2. What is the territory from which the users come?

3. What is the socioeconomic condition of the users?

4. Age range, gender

5. Is there a predominance of any type of drug disorder?

6. Is there any data on dropout rate / number of discharges due to improvement / user turnover?

BLOCK 2. ABOUT HIS PARTICIPATION IN THE GROUP

7. How do you evaluate your participation overall?

8. Benefits? Disadvantages?

9. What was it like to participate with the patients?

BLOCK 3. ABOUT THE IMPLEMENTATION OF MBRP IN THE SERVICE

10. How do you evaluate the implementation of MBRP here in the service?

11. How was the conversation / mobilization process with the team?

12. Did it impact the service in any way (team, structuring of activities, etc.)? Positively and negatively.

13. How were participants recommended for MBRP?

14. What was the reaction (acceptance / myths / refusal...) of the patients when MBRP was offered?

15. Was it possible to observe any impact, positive or negative, of MBRP on CAPS-ad patients?

16. In your opinion, would professionals be interested in undergoing training to apply MBRP?

BLOCK 4. ABOUT THE IMPLEMENTATION OF MBRP IN THE SERVICE

17. Does it make sense to conduct MBRP here at CAPS-ad? Does it align with the service's care proposal, culture, or objectives? Would it be of interest to the institution's professionals?

18. If yes, what would be necessary for implementation? What would hinder or facilitate it?

19. Is it suitable for the service's infrastructure or activity schedule?

3 Semi-structured script of the in-depth interview with professionals

BLOCK 1. GENERAL CHARACTERISTICS OF THE SERVICE

1. Coverage area - what is it and what are the socio-economic characteristics?

2. Management form(s)

3. Population coverage

4. CAPS-ad type II or III

5. Professionals who make up the team (who? How many?). Is there turnover?

BLOCK 2. GENERAL CHARACTERISTICS OF THE SERVICE USERS:

6. What is the territory from which the users come?

7. What is the socio-economic condition of the users?

8. Age range, gender

9. Is there a predominance of any type of drug disorder?

10. User entry method into the service

11. Is there any data on dropout rate / number of discharges due to improvement / user turnover?

BLOCK 3. GENERAL CHARACTERISTICS OF THE OFFERED SERVICES

12. Activities and services offered

13. Do all users participate in all activities? How is participation defined?

14. What is the service's care flowchart (arrival, reception, screening...)?

15. How long do users stay in the services (full-time? Intensive, semi, non-intensive?)

16. What are the user discharge methods?

BLOCK 4. ABOUT THE IMPLEMENTATION OF MBRP IN THE SERVICE

17. How do you evaluate the implementation of MBRP here in the service?

18. How did the conversation / team mobilization process go?

19. Did it impact the service in any way (team, activity structuring, etc.)? Positively and negatively.

20. Can you say what the patient reaction was (acceptance / myths / refusal...) when MBRP was offered?

BLOCK 5. ABOUT THE IMPLEMENTATION OF MBRP IN THE SERVICE

21. Does it make sense to conduct MBRP here at CAPS-ad? Does it align with the service's care proposal, culture, or objectives? Would it be of interest to the institution?

22. If yes, what would be necessary for implementation? What would hinder or facilitate it?

23. Is it suitable for the service's infrastructure or activity schedule?
