# Supplementary material for: A mindfulness-based intervention for Substance Use Disorder in a Brazilian vulnerable population: a feasibility mixed method study
Source: Front Public Health. 2024 Oct 30;12:1381489. doi: 10.3389/fpubh.2024.1381489 (PMC11557387; doi:10.3389/fpubh.2024.1381489)
Supplement: Supplementary file 2 [file Table_1.pdf]

Supplementary Material

MBRP for Substance Use Disorder in a Brazilian Vulnerable Population: A Feasibility Mixed Method Study

Mayra Pires Alves Machado[1], Emérita Sátiro Opaleye[1*], Andre Bedendo[1,2], Sarah Bowen[3], Ana Regina Noto[1]

[1] Núcleo de Pesquisa em Saúde e Uso de Substâncias, Departamento de Psicobiologia, Universidade Federal de São Paulo, São Paulo, Brazil

[2] Department of Health Sciences, University of York, York, UK.

[3] School of Graduate Psychology, Pacific University, Forest Grove, OR, USA

*** Correspondence:**
Corresponding Author
emerita.satiro@unifesp.br

1 Supplementary Figures and Tables

Supplementary Table: Benefits reported regarding behavioral and emotional outcomes

Inhibit risk	Expand protection	
• •Reduction of interpersonal conflicts<br>• Decrease or control of aggression<br>• Reduction of anxiety symptoms<br>• Reduction of stress and nervousness	• Aspects related to serenity (calmness, tranquility, tolerance, and patience)<br>• Improvement in interpersonal relationships	• Routine planning<br>• Increase in the repertoire of healthy and meaningful activities (e.g., religion, diet, physical activity, etc.), with greater self-care

• Decrease in irritation<br>• Reinterpretation of triggers (no longer bothersome)<br>• Distancing from third-party problems<br>• Reduction of concerns about the future	• Enhancement of self-confidence, self-esteem, and self-efficacy<br>• Improvement in sleep<br>• Increase in the repertoire of assertive responses	• Aspects related to well-being (peace, balance, gratitude, and joy in life)<br>• Feeling "alive"
